# Outcomes of Mini-Invasive Arthroscopic Arthrolysis Combined with Locking Screw and/or Intramedullary Nail Extraction after Osteosynthesis of the Proximal Humerus Fracture

**DOI:** 10.3390/jcm11020362

**Published:** 2022-01-12

**Authors:** Roman Madeja, Jana Pometlová, Roman Brzóska, Jiří Voves, Lubor Bialy, Leopold Pleva, Jan Stránský, Adéla Vrtková, Jaroslav Janošek, Kristýna Čabanová

**Affiliations:** 1 Department of Trauma Surgery, University Hospital Ostrava, 17. Listopadu 1790, 708 52 Ostrava, Czech Republic; jana.pometlova@fno.cz (J.P.); Jiri.voves@fno.cz (J.V.); lubor.bialy@fno.cz (L.B.); leopold.pleva@fno.cz (L.P.); Jan.stransky@fno.cz (J.S.); 2 Faculty of Medicine, Institute of Emergency Medicine, University of Ostrava, Syllabova 19, 703 00 Ostrava, Czech Republic; 3 Department of Orthopedics, St. Luke’s Hospital, Bielsko-Biała, Bystrzańska 94b str., 43-300 Bielsko-Biała, Poland; rbrzoska13@gmail.com; 4 Department of Applied Mathematics, Faculty of Electrical Engineering and Computer Science, VSB—Technical University of Ostrava, 708 00 Ostrava, Czech Republic; adela.vrtkova@vsb.cz; 5 Department of the Deputy Director for Science and Research, University Hospital Ostrava, 17. Listopadu 1790, 708 52 Ostrava, Czech Republic; 6 Center for Health Research, Faculty of Medicine, University of Ostrava, Syllabova 19, 703 00 Ostrava, Czech Republic; janosek@correcta.cz; 7 Centre for Advanced Innovation Technologies, VŠB—Technical University of Ostrava, 708 33 Ostrava, Czech Republic; kristina.cabanova@vsb.cz; 8 Faculty of Mining and Geology, VŠB—Technical University of Ostrava, 708 33 Ostrava, Czech Republic

**Keywords:** proximal humerus fracture, intramedullary nail, shoulder, arthroscopy, extraction, screw, Constant–Murley shoulder score, arthrolysis, post-operative dysfunction

## Abstract

Data on the effectiveness of arthroscopic arthrolysis and extraction of osteosynthetic material after osteosynthesis of the proximal humerus in patients with persisting problems are rare and insufficient. In this study, we performed arthroscopic arthrolysis and extraction of fixation screws, and, where protruding, extraction of the nail in 34 patients with problems persisting 12 months after osteosynthesis of the proximal humerus using an intramedullary nail. The effectiveness of the treatment was assessed using the Constant–Murley shoulder score and forward flexion difference between the treated arm and the contralateral one. A median increase of 16 points in CMS score and 30 degrees reduction in the arm forward flexion difference was recorded 12 months after the arthroscopy. The improvement was significantly higher in the patient group with intramedullary nail extraction (however, this group had worse pre-operative values and the screw was only extracted where likely to cause problems). The median time to heal was 11 weeks; no serious peri- or post-procedural complications occurred. Mini-invasive arthroscopic arthrolysis combined with extraction of osteosynthetic material proved to be a safe and effective method for treatment of patients after osteosynthesis of the proximal humerus using an intramedullary nail with persisting pain and/or mobility limitation.

## 1. Introduction

The development of modern implants ensuring stable osteosynthesis of the proximal end of the humerus facilitating early rehabilitation has led to an increase in the number of such osteosyntheses [1]. This approach led to an improvement of the outcomes in the treatment of such fractures and improvements in further joint functionality. Two most common methods can be distinguished—plate osteosynthesis and intramedullary nail osteosynthesis; other methods, such as Kirschner wires, individual screws, etc. are used to a lesser extent [2]. Each of such methods has its pros and cons and are better suited for certain types of injuries; in a recent systematic review and meta-analysis, however, osteosynthesis by intramedullary nail has been shown to be superior to that by locking plate [3].

Nevertheless, even after the fracture has healed and the patients have undergone rehabilitation, many still suffer from strain pain or even pain at rest, as well as from continued limited mobility of the shoulder joint [4,5], which significantly negatively influences their quality of life and prevents them from performing common everyday activities (e.g., putting various items on shelves, lifting items, etc.). To improve the patients’ conditions, the osteosynthetic material is often surgically extracted, sometimes with redress of the joint and subsequent rehabilitation; the effectiveness of such an approach is, however, often suboptimal [3]. The likely cause is that the new open surgery represents new major stress, and, despite possible redress, new adhesions can form during wound healing.

The development of arthroscopic techniques facilitated more accurate diagnosis in the region of the shoulder joint [6] but also brought about the option of mini-invasive disruption of the fibrous adhesions (arthrolysis) and even mini-invasive extraction of the osteosynthetic material, which should be gentler than open surgery [7]. The perioperative burden to the organism is minimal and so are the post-procedural complications [8]. 

Studies focusing on arthroscopic extraction of osteosynthetic material after the osteosynthesis of the proximal end of the humerus are rare and are limited usually either by a small number of patients [9] or by the low homogeneity of the patient group (for example, various implant types and fractures) [10].

Hence, we decided to evaluate the outcomes of the mini-invasive arthroscopic arthrolysis combined with the extraction of the osteosynthetic material in patients after the osteosynthesis of the proximal humerus by an intramedullary nail in patients with a shoulder mobility deficiency and/or pain persisting more than 12 months after the surgery. We hypothesized that in such patients, the arthroscopic arthrolysis with or without nail/screw extraction will (i) improve the shoulder mobility, (ii) reduce pain, and (iii) improve the patient’s quality of life as indicated by the CMS. Furthermore, we hypothesized that (iv) the improvement will be greater in the group in which nail extraction was performed than in the group with arthroscopic arthrolysis only.

## 2. Materials and Methods

### 2.1. Patient Group and Inclusion/Exclusion Criteria 

This retrospective study analyzed data of patients treated between 2012–2017 at the Trauma Department at the University Hospital Ostrava, who underwent osteosynthesis of the proximal end of the humerus by a straight intramedullary proximal humeral nail with proximal and distal locking screws (Targon^®^ PH, B Braun, Melsungen, Germany) according to the AO Foundation guidelines for low-invasive proximal humeral nailing by the anterolateral approach [11], which can be employed also for nailing of four-part fractures [12]. All humeral fractures included in this study were classified by the surgeons performing the original osteosyntheses as intra-articular and/or four-part fractures 11C according to the AO/OTA classification 2018 [13]. It is necessary to emphasize that the presented study does not analyze or discuss the methods and outcomes of primary osteosynthesis but only outcomes of a follow-up arthroscopic arthrolysis with extraction of the osteosynthetic material. The study was approved by the Ethics Committee of the University Hospital Ostrava No. 1100/2021. All patients included in the study signed informed consent with anonymous use of their data in retrospective studies during their hospital stay.

We evaluated the outcomes of the arthroscopic procedure in patients with problems persisting 12 months after the original surgery. The patient group contained no patients with malunion, i.e., dislocation between fragments of more than 5 mm and/or axial dislocation of more than 20 degrees, or nonunion of the fracture as such patients were not referred for arthrolysis; therefore, in view of the retrospective character of the study, they were not present in the analyzed group. The inclusion criteria were as follows: (i) significantly reduced mobility of the shoulder joint (forward flexion reduced by ≥30 degrees compared to the contralateral “healthy” side) and/or inferior overall results (Constant–Murley shoulder score, CMS, <85 points) 12 months after the original proximal humerus osteosynthesis with intramedullary nail, and (ii) the progress over the last 6 months not exceeding 5% in the CMS score and 5 degrees in the forward flexion despite taking physiotherapy. 

Exclusion criteria were osteonecrosis of the head of the humerus, neurological defect, marks of rotator arthropathy, and damage to the rotator cuff requiring surgical suture during the primary injury—as in these cases, arthrolysis and extraction of the nail are unlikely to improve the outcome. Other exclusion criteria comprised injury or another pathology in the region of the contralateral shoulder joint (which would make the comparison of forward flexion difference between shoulders impossible) and X-ray-confirmed osteosynthetic material protruding by more than 5 mm (in which case the nail protrusion would represent the main problem, limiting the motion due to its interference with the surrounding bone tissue and causing pain; in such cases, the effect of nail removal would be much greater than that of arthrolysis itself, which would bias the comparison with the group without nail extraction). 

These criteria ensured that only patients in whom further conservative treatment would yield no further improvement (as it led to no improvement over the last 6 months) were included in this study and the patients’ condition at that time point could serve as a point of reference for the comparison of further progress resulting from the subsequent treatment as described below. 

In all, osteosynthesis was performed in 247 patients over the study period, of which 168 were treated using the intramedullary nail. Of these, 34 patients met the inclusion and exclusion criteria. The descriptive characteristics of this group are summarized in Table 1. No statistically significant difference in age between men and women was detected (*p* = 0.705). 

### 2.2. Arthroscopic Arthrolysis and Osteosynthetic Material Extraction 

**Figure 1:**
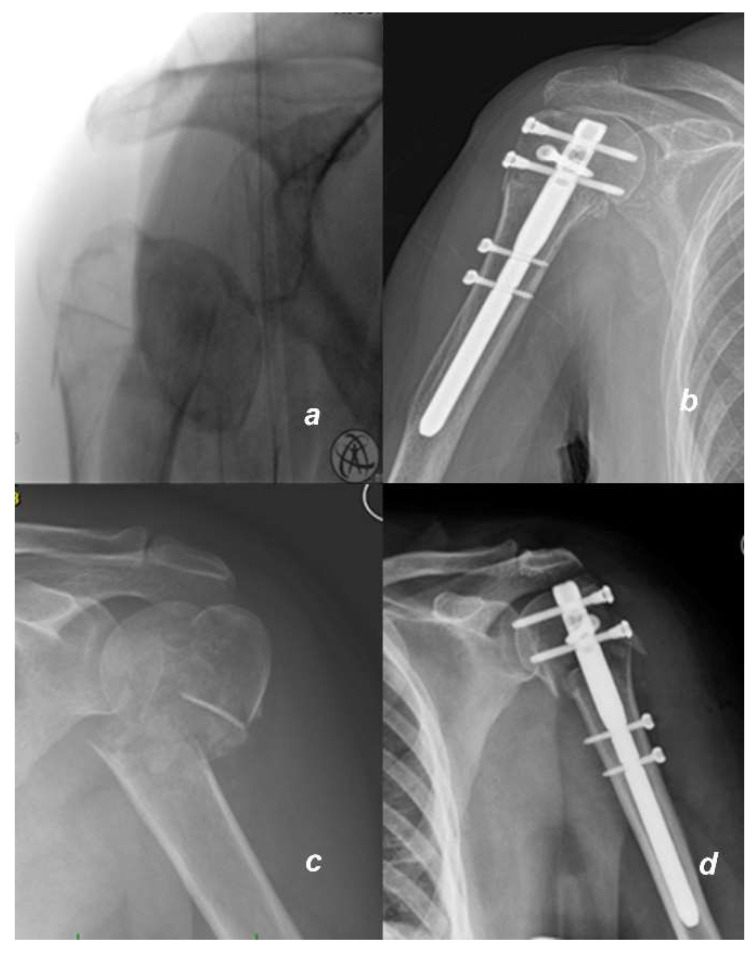
X-rays of fractures and osteosyntheses in two patients: (**a**) Fracture of proximal humerus in a 57-years-old man with intraarticular fracture 11C1.1 (AO) before and (**b**) 12 weeks after osteosynthesis with humeral intramedullary nail; as the nail is not protruding, only proximal locking screws would be removed during arthrolysis; (**c**) a 65-year-old woman with a proximal humerus fracture 11C3.1 before and (**d**) 6 weeks after osteosynthesis; the nail is protruding. All screws including the nail would be removed during arthrolysis.

**Figure 2:**
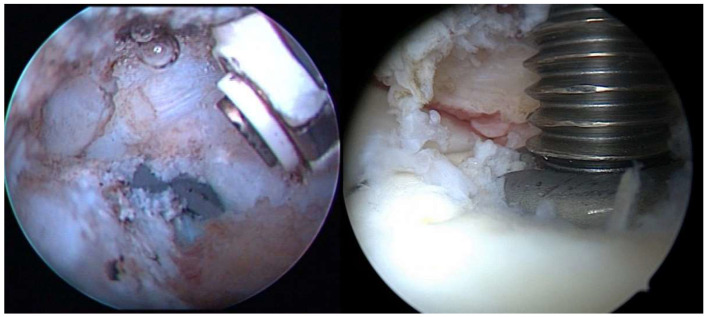
Arthroscopic imagery. (**left**) Fibrous adhesions between the tendon of the supraspinatus muscle and the humeral head with a partially protruding end of the nail; (**right**) extraction of the protruding nail under arthroscopic control.

In our department, shoulder joint arthroscopy with arthrolysis and extraction of the fixation screws is the standard method of treatment of such patients; if the intramedullary screw itself protrudes out of the bone, it is also extracted during the procedure (Figure 1). The procedure is always performed by the same surgeon in our department, who specializes in this type of operation, and it is performed as follows:

Under general anesthesia in the beach chair position, the subacromial space is penetrated through the lateral and anterosuperior arthroscopic entry to search for fibrous adhesions from the previous surgery. Such adhesions are removed using the VAPR™ device (DePuy Synthes, Raynham, MA, USA) or shaver. Subsequently, we penetrate into the glenohumeral joint through the posterior and anterosuperior arthroscopic entry and search for fibrous adhesions between the humeral head and the rotator cuff; any detected adhesions are removed. If the nail is prominent above the level of the humeral head cartilage, we remove the nail and the locking screws (in this study, due to the exclusion criteria, only nails protruding 1–5 mm were extracted; see exclusion criteria). If the nail is embedded, only proximal locking screws from the humeral head, which may also cause pain and limited mobility, are extracted (see Figure 2).

The procedure is followed by physiotherapy with regular follow-ups; during which, indicators of the outcome (difference in forward flexion compared to the other side and Constant–Murley shoulder score, CMS) are recorded. CMS is a scoring system evaluating the subjective outcome of the treatment, activities of daily living, pain, and the mobility of the shoulder in all planes. Its four components (pain—the maximum 15 points = no pain; activities of daily living—maximum 20; range of motion—maximum 40; strength—maximum 25) serve for evaluation of the quality of the patient’s life [14]. A limitation of this scoring system is that the mobility of the joint is not compared to the other side; in other words, if the patient’s mobility of both shoulder joints is impaired even before the injury, the CMS cannot reach the maximum value, regardless of the success of the procedure. Despite this limitation of the CMS, it is one of the most widely used scoring systems for evaluation of the shoulder function. Nevertheless, to account for this limitation, we used an additional parameter of the forward flexion difference between both shoulders, which provides a more objective evaluation of the most important type of shoulder motion. Please note that numerically, the reduction of forward flexion difference between shoulders is equivalent to forward flexion improvement in the operated arm; the advantage of evaluating the difference between shoulders lies, as mentioned above, in a better indication of the surgery success (for example, if the patient’s maximum forward flexion of the uninjured shoulder is, for example, 160 degrees, we cannot reasonably expect the arthrolysis to yield a better result than that). 

### 2.3. Data Analysis

For the purposes of this study, we analyzed the data from the 12-month follow-up visit. In addition, we also recorded the time to heal, i.e., the time after which the patient’s condition ceased to change (improve) further. We analyzed the overall change in the forward flexion difference between shoulders and CMS score (a) in the entire patient group and (b) separately for groups that did or did not undergo nail extraction.

Numerical variables are expressed as medians and interquartile range (IQR, lower and upper quartile). The categorical variable (sex) is presented as absolute frequencies and relative frequencies in percentages. Defined groups were compared using the Chi-square test of independence for contingency tables (sex) or the Mann–Whitney test (all other variables). The significance of the progress of selected parameters in time was tested by the paired Wilcoxon test and visualized with paired boxplots. All statistical analyses were performed using R software (version 4.0.2, www.r-project.org, accessed on 30 June 2021) and the significance level was set to 0.05. Power analysis was not performed for this retrospective study as all eligible patients were included in the study.

## 3. Results

The basic patient group characteristics are detailed in Methods. The median time to heal in the entire group was 11 weeks (interquartile range of 9 to 14). No serious complications or infectious complications were observed during or after the procedure. 

**Figure 3:**
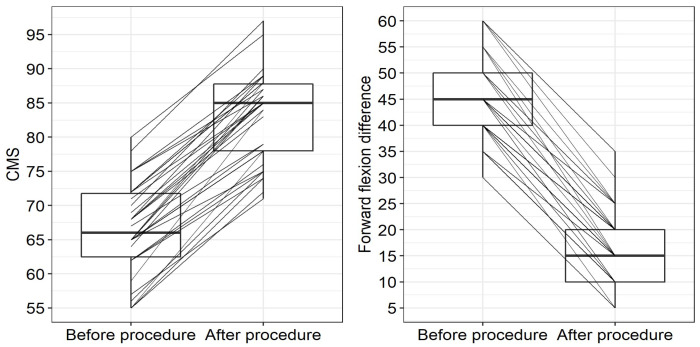
Constant–Murley score (CMS)—(**left**)—and forward flexion difference—(**right**)—between the individual patients’ conditions before and after the arthroscopic arthrolysis and osteosynthetic material extraction—boxplots including paired results of individual patients; please note that while positive change signifies an improvement in CMS, negative change indicates improvement in the parameter “Forward flexion difference between shoulders”.

A statistically significant improvement of the patients’ conditions were observed 12 months after the arthroscopic procedure (see Table 2) in all studied parameters including all individual components of the CMS score. A significant CMS improvement (*p* < 0.001) was observed with a median increase of 16 points (IQR: 13–19; see also Figure 2 on the left for individual records). Similarly, a significant improvement in the forward flexion difference between the treated and contralateral shoulder was observed after the arthroscopic procedure, with the median reduction in the forward flexion difference of 30 degrees (IQR: 25–30 degrees; see Figure 3 on the right for individual records). Hence, arthrolysis significantly improved the condition of all patients in our group (see Figure 2); note that in all these patients, the condition stagnated for at least 6 months before the procedure when using conservative treatment.

As an additional analysis, we compared groups of patients in whom the intramedullary nail itself was or was not extracted during arthrolysis (please note that locking screws were extracted and arthrolysis performed in all patients within the study group). The comparison of the outcomes in the “no nail extraction” (NNE) and “nail extraction” (NE) groups is presented in Table 3. The improvement in the CMS value was significantly higher (*p* = 0.001) in the NE group (median improvement 19 points; IQR: 16–21) than in the NNE group (median improvement 14 points; IQR: 13–17). Before the arthroscopic procedure, the difference between the groups was borderline insignificant (*p* = 0.056) while 12 months after the procedure, the lack of difference between groups caused by significant improvement in both groups was obvious (*p* = 0.804).

A statistically significant difference (*p* < 0.001) was also observed when comparing the NNE and NE groups from the perspective of the forward flexion difference between arms; the forward flexion difference between the shoulder joints was significantly more reduced (i.e., the improvement was greater) in the NE group (median 30 degrees; IQR: 30–35 degrees) than in the NNE group (median 25 degrees, IQR: 25–30 degrees; *p* < 0.001). This corresponds to the finding that preoperatively, the difference in the forward flexions between sides was higher in the NE group than in the NNE group (*p* = 0.019); while after the arthroscopy, there was no difference between groups (*p* = 0.985), see Table 3.

It is also necessary to note that the time to heal after the arthroscopic arthrolysis (i.e., the time after which the final improvement is achieved), was relatively short (a median of 11 weeks).

## 4. Discussion

In this retrospective study, we evaluated the effect of mini-invasive arthrolysis combined with locking screw and, where applicable, intramedullary nail extraction, in patients after the proximal humerus osteosynthesis by intramedullary nail in whom problems persisted more than 12 months after the osteosynthesis and failed to improve over the last 6 months before arthrolysis. Particularly important information, which further supports our findings, is that the CMS improvement was not due to a major improvement in a single parameter accompanied by stagnation in the remaining ones; instead, an overall significant improvement in all components of the CMS score was observed (see Table 2). We can, therefore, safely state that the arthrolysis accompanied with the extraction of the proximal locking screws and, where justified, of the nail, had a significant positive effect in the selected group of patients.

Performing arthroscopy aiming at improving the patient’s condition after the osteosynthesis of the proximal humerus has been reported previously in the literature. However, many such papers simply describe the technique used [15,16] or have a small patient group [9]. Also, most of these studies describe arthrolysis and LCP (locking compression plate) extraction but do not deal with intramedullary nail extraction or, generally, secondary surgery after intramedullary nail osteosynthesis. For, example, Dines et al. [17] and Voigt et al. [18] described arthroscopy-assisted LCP extraction as a mini-invasive technique with good results supporting also the examination of the joint and, if necessary, intervention on the adhesions formed in the shoulder joint. Katthagen et al. performed arthroscopy in 45 patients after osteosynthesis of the proximal humerus using LCP and found damage of the cartilage, rotator cuff, or tendon of the biceps in 75% of patients. In accordance with our study, they detected a significant improvement in the CMS and mobility of the shoulder joint after the arthroscopic intervention [19]. Another study by Katthagen et al. compared the outcomes of arthroscopy-assisted mini-invasive plate extraction after osteosynthesis of the proximal humerus (20 patients) to the outcomes of open extraction (9 patients), proving the significant benefits of the mini-invasive approach [20].

Good results of arthroscopic arthrolysis of the shoulder joint after osteosynthesis using plates were also reported by Maqdes et al., who registered CMS improvement by 17.1 points after the arthroscopic arthrolysis of the shoulder joint [21]; the results are similar to ours (median improvement of 16 points). A recent multicenter study of 58 patients describes arthroscopy of the shoulder joint with arthrolysis and extraction of osteosynthetic material (plates and nails) leading to a significant improvement of patients’ conditions [10]. Median CMS improvement in that study was 32 points, which is significantly more than in our study; however, the pre-operative CMS values in their study were lower than in our study, leaving a bigger space for improvement; besides, the group included patients with various types of fractures, the indication criteria for osteosynthesis included three different problems, and osteosynthesis by nail was performed only in 25 patients in their group. Therefore, although the results are valuable, we cannot consider this group homogeneous enough. The only other study investigating the effects of arthroscopic extraction of humeral nails was that by Kim et al., who emphasized the benefits of this approach [9]; their study, however, contained only six patients. 

To the best of our knowledge, therefore, no study reporting the arthroscopic extraction of the intramedullary nail in a patient group as big as ours has been published so far. Besides, the available studies on shoulder arthroscopy usually describe only the shoulder mobility and, unlike in our study, they do not compare the forward flexion of the injured arm to the contralateral (“healthy”) side [22,23,24]. The approach used in our study provides much better information about the success of the treatment than simple forward flexion as the latter can vary even without even the effects of the injury (depending on the patient’s age, sex, and general health condition). 

The improvements of patients’ conditions were significantly greater in the patient group where the intramedullary nail was extracted in addition to the arthrolysis and removal of proximal locking screws than in the group in which only the latter two procedures were performed. Rather than being able to recommend nail extraction in all patients, however, we believe that this is likely to be caused by the generally worse initial condition of the patients in whom the nail was extracted—it was extracted only in patients in whom it obviously protruded and was likely to cause irritation or pain. Hence, the greater improvement in patients after nail extraction is not a surprise as they had both greater space for improvement and an obvious causative agent was removed.

Another interesting piece of information is the time to heal, which was defined as the time after which no further improvement of the forward flexion/CMS occurred. In our study, the median time to heal was 11 weeks, which is, in view of the significant improvement of the patients’ condition, a relatively short time; this is especially true from the perspective that the patients were referred for the arthroscopic procedure only after 12 months from the original surgery, when their condition was no longer improving despite conservative therapy. 

### Study Limitations

Some issues that could be perceived as limitations should be explained. First, it may appear that this study lacks the traditional control group. However, it must be taken into account that a failure of conservative treatment (i.e., no significant change in condition over the last 6 months despite physiotherapy) was a prerequisite for the inclusion of the patient into the study. For this reason, we can consider the baseline values before the procedure as values after exhausting the potential of conservative treatment and we can safely make comparisons and draw conclusions regarding the effectiveness of arthrolysis from this baseline. Secondly, with the exception of conservative treatment as explained above, we do not compare the results of arthrolysis to results of other methods (such as open surgical screw/nail extraction) or discuss arthrolysis after other methods of osteosynthesis. At our department, we do not perform open surgical extraction where it can be avoided and prefer a mini-invasive solution, which is in accordance with the current trends in medicine. The principal drawback of open surgical arthrolysis and nail extraction lies in the high degree of invasiveness, which is associated with a prolonged convalescence period and, moreover, has a greater potential for infectious complications and the development of new adhesions [20,25]. As intramedullary nail extraction can be performed mini-invasively, we did not have any open surgical extractions in our study group. To maintain the homogeneity of the study group, we did not include any patients in whom osteosynthesis was performed using other methods (Kirschner wires, cannulated screws, LCP), either.

Of course, the retrospective single-center design, associated with the relatively small number of patients, could be also perceived as a limitation to our study. Nevertheless, the number of patients in our study is comparable with the largest studies in the field even where secondary arthroscopic techniques after other methods of primary osteosynthesis, such as locking plates, are concerned. If only intramedullary nails are taken into consideration, our patient group is the largest by far. In addition, the homogeneity of our patient group can be considered a significant strongpoint of our study, as well as the evaluation of the treatment outcome (forward flexion compared to the contralateral, uninjured, shoulder). 

In a way, this homogeneity can be at the same time considered a downside as, although it allows us to make our claims with a high degree of certainty, it limits the range of patients in whom we can claim it to be beneficial. It is, however, a starting point opening the gates for further investigations in the field, which should lead to the increase in the use of this method also for treatment of patients with problems persisting after nail osteosyntheses of other humeral fractures. 

This method has for a long time almost exclusively been used for the treatment of such patients at our hospital and provides, as is obvious from the results reported in this study, major improvement in the clinical condition of such patients. For this reason, there are ethical considerations that would render a prospective design of such a study, which would be associated with denying this method to a number of such patients, practically impossible. In view of this fact, a retrospective design is the only one eligible at our hospital for evaluation of the success of this method. Nevertheless, the strengths of our study (group homogeneity, relatively large patient group) make the results valid despite these shortcomings.

## 5. Conclusions

Our results demonstrate that the mini-invasive arthroscopic arthrolysis of the shoulder joint combined with the removal of fixation screws and (where needed) the intramedullary nail is a great method for improving the results of osteosynthesis after the proximal humerus fracture using intramedullary nail in patients with persisting problems. The presented study demonstrates this on a homogeneous group of 34 patients with 11C fractures treated by intramedullary nails; however, it is likely that the same method can successfully help in the treatment of patients with other proximal humeral fractures treated with intramedullary nails, in whom the original osteosynthesis yielded suboptimal results.

## Figures and Tables

**Table 1:** Basic characteristics of the patient groups (all patients, patients in whom no nail extraction was performed—NNE, and patients in whom nail was extracted—NE); *p*-value indicates the significance of differences between the NNE and NE GROUPS.

	Median (IQR) or *n* (%) ^a^			
	Total (*n* = 34)	NNE (*n* = 21)	NE (*n* = 13)	*p*
Age (years)	58 (48; 64)	58 (48; 62)	58 (52; 65)	0.467 ^b^
Sex (male)	22 (65%)	15 (71%)	7 (54%)	0.501 ^c^

^a^ The median and interquartile range (age; lower and upper quartile) or the absolute and relative frequency in percentages (sex); ^b^ the *p*-value of the Mann–Whitney test; ^c^ the *p*-value of the Chi-square test of independence.

**Table 2:** Group characteristics, overall outcomes of the treatment and effects of treatment on individual components of the Constant–Murley score.

	Median (IQR) ^a^			
	Before Procedure	After Procedure	Improvement	*p* ^b^
Forward flexion difference (degrees)	45 (40; 50)	15 (10; 20)	30 (25; 30)	<0.001
CMS (max. 100 = no limitation)	66 (62; 72)	85 (78; 88)	16 (13; 19)	<0.001
Pain (max. 15)	10 (10; 15)	15 (15; 15)	5 (5; 5)	<0.001
Activities of daily living (max. 20)	15 (14; 16)	17 (16; 18)	2 (1; 3)	<0.001
Strength (max. 25)	13 (10; 15)	15 (14; 17)	2 (2; 3)	<0.001
Range of motion (max. 40)	28 (25; 30)	35 (32; 38)	6 (4; 10)	<0.001

^a^ The median and interquartile range (lower and upper quartile) or the absolute and relative frequency in percentages; ^b^ the *p*-value of the paired Wilcoxon test.

**Table 3:** Comparison of group characteristics and treatment outcomes of patients in whom the intramedullary nail was (NE group) or was not (NNE group) extracted during the arthrolysis (please note that proximal locking screws were removed and arthrolysis performed in all patients).

	Median (IQR) ^a^		
	NNE (*n* = 21)	NE (*n* = 13)	*p* ^b^
**CMS**			
Before procedure	68 (65; 72)	65 (59; 68)	0.056
After procedure	85 (78; 87)	84 (78; 88)	0.804
Improvement	14 (13; 17)	19 (16; 21)	0.015
**FFD (degrees)**			
Before procedure	40 (40; 45)	50 (45; 55)	0.019
After procedure	15 (10; 20)	15 (10; 20)	0.985
Improvement	25 (25;30)	30 (30; 35)	<0.001
Time to heal	9 (8; 12)	12 (11; 14)	0.020

^a^ The median and the interquartile range (lower and upper quartile) or the absolute and relative frequency in percentages; ^b^ the *p*-value of the Mann–Whitney test or the Chi-square test of independence; FFD = forward flexion difference between shoulders. CMS–Constant-Murley score.

## Data Availability

Data are available from Figshare at https://doi.org/10.6084/m9.figshare.14922975.v2 (posted on 7 July 2021).
